# Sequencing of cancer cell subpopulations identifies micrometastases in a bladder cancer patient

**DOI:** 10.18632/oncotarget.17312

**Published:** 2017-04-21

**Authors:** Kris Prado, Kelvin X. Zhang, Matteo Pellegrini, Arnold I. Chin

**Affiliations:** ^1^ Department of Urology, UCLA, Los Angeles, CA 90095, USA; ^2^ Merck Sharp & Dohme Co., Computational Genomics and Informatics, Boston, MA 02210, USA; ^3^ Department of Molecular, Cell, and Developmental Biology, UCLA, Los Angeles, CA 90095, USA; ^4^ Broad Stem Cell Research Center, UCLA, Los Angeles, CA 90095, USA; ^5^ Jonsson Comprehensive Cancer Center, UCLA, Los Angeles, CA 90095, USA

**Keywords:** bladder cancer, next-generation sequencing, micrometastases, cancer initiating cells, pathologic staging

## Abstract

**Purpose:**

Pathologic staging of bladder cancer patients remains a challenge. Standard-of-care histology exhibits limited sensitivity in detection of micrometastases, which can increase risk of cancer progression and delay potential adjuvant therapies. Here, we sought to develop a proof of concept novel molecular approach to improve detection of cancer micrometastasis.

**Experimental Design:**

We combined fluorescence activated cell sorting and next-generation sequencing and performed whole-exome sequencing of total cancer cells and cancer cell subpopulations in multiple tumor specimens and regional lymph nodes in a single patient with muscle-invasive urothelial carcinoma of the bladder following radical cystectomy.

**Results:**

Mean allele frequency analysis demonstrated a significant correlation between primary tumor cancer cells and cancer cells isolated from the lymph nodes, confirming lymph node disease despite negative pathologic staging. RNA-sequencing revealed intratumoral heterogeneity as well as enrichment for immune system and lipid metabolism gene sets in the micrometastatic cancer cell subpopulations.

**Conclusions:**

Our analysis illustrates how next-generation sequencing of cancer cell subpopulations may be utilized to enrich for cancer cell markers and enhance detection of bladder cancer micrometastases to improve pathologic staging and provide insight into cancer cell biology.

## INTRODUCTION

Latent recurrences despite favorable pathology has been a longstanding conundrum in surgical oncology. In patients with muscle-invasive urothelial carcinoma of the bladder, radical cystectomy and pelvic lymph node dissection provides local surgical control. Despite complete resection based on traditional gross and histological staging, patients with clinically localized muscle-invasive urothelial bladder carcinoma (pT2 N0) have a recurrence rate of 11-35% following radical cystectomy, with recurrence rates in higher stages of localized disease up to 50-69% (pT4 N0) [1]. Recent advances in immunotherapy combined with systemic cisplatin-based chemotherapy have provided additional options for adjuvant therapy [2, 3]. As bladder cancer progresses based on a predictable pattern of lymphatogenous metastasis with recurrence attributed to lymph node micrometastases, improved pathologic staging of lymph nodes may add precision to selecting adjuvant therapy as well as stratifying patients with metastatic disease [4].

Cancer stem cells (CSC) have been implicated as a source of early metastasis and disease progression [5]. They represent a self-renewing and pluripotent population that can reconstitute all cell types of an individual tumor. Enrichment of stem-like genes has been observed in single cell analysis of early, low-volume metastases in an *in vivo* mouse model for breast cancer as well as in highly aggressive small cell neuroendocrine prostate cancer [6, 7]. In bladder cancer, the surface markers CD44 and CD49f have been implicated in the cancer-initiating population important in cancer self-renewal and repopulation [8–10]. Therefore, we postulated that bladder cancer CD44^+^CD49f^+^ cells may be a critical component of metastasis, and hypothesized that characterization of these cancer cell subpopulations from multiple tumor regions and lymph nodes will identify micrometastases as well as molecular profiles that may elude to metastatic potential. Here, we prospectively purified and characterized total and EPCAM^+^CD44^+^CD49f^+^ subpopulations in fresh, surgically-resected tissue from multiple regions of a bladder tumor and regional lymph nodes by whole-exome sequencing and RNA-sequencing in a single patient with clinically localized muscle-invasive urothelial bladder carcinoma.

## RESULTS

**Figure 1:**
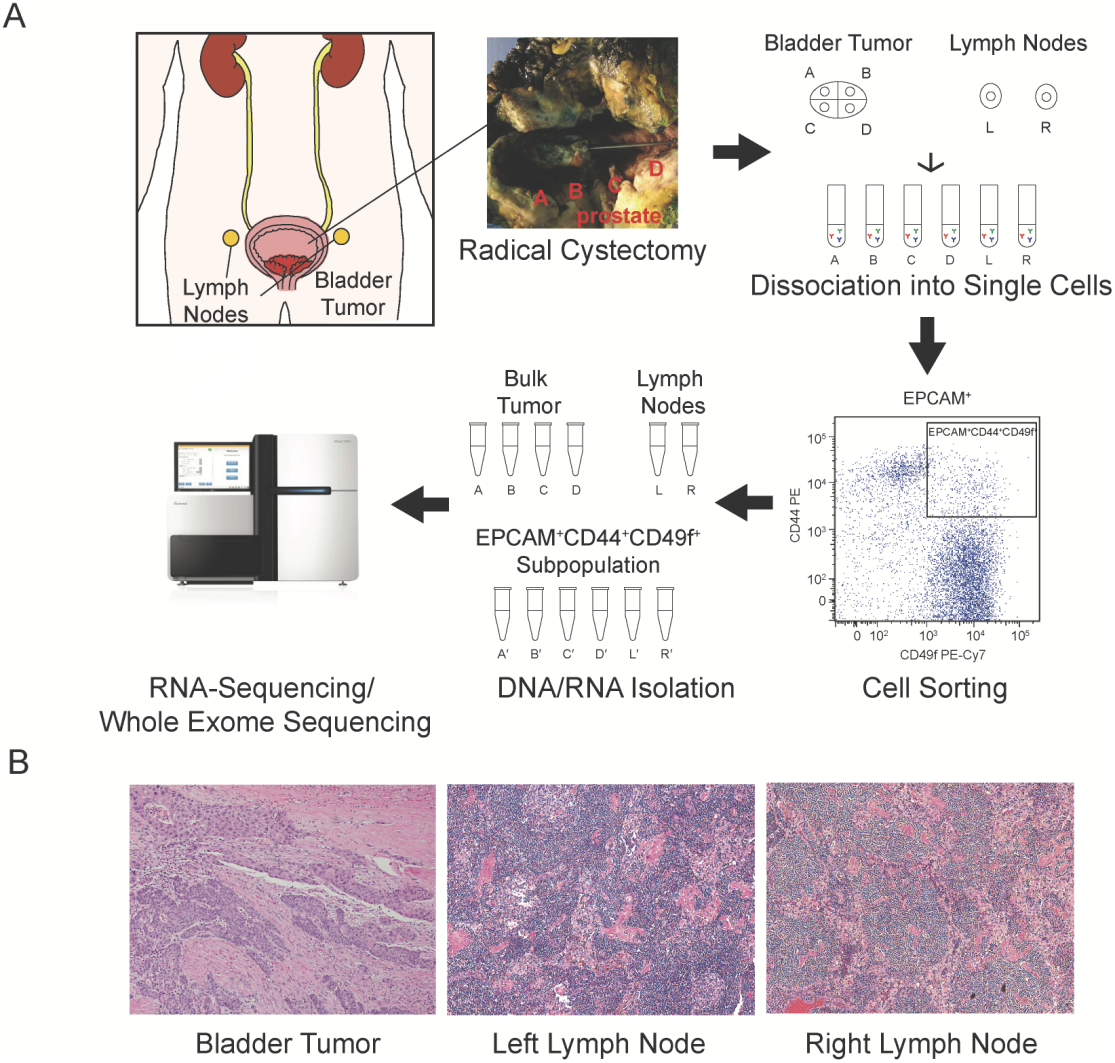
Analysis schematic Radical cystoprostatectomy and extended pelvic lymph node dissection were performed, and fresh tissue from four regions of the bladder tumor and representative left and right lymph nodes were dissociated into single cell suspensions and fluorescence activated cell sorting isolated the EPCAM^+^CD44^+^CD49f^+^ cancer cell subpopulation. DNA and RNA were isolated from the bulk tumor regions (samples A-D) and lymph nodes (samples L and R) and the corresponding cancer cell subpopulations (denoted with′) and utilized for whole-exome sequencing and RNA-sequencing **(A)**. Representative histology of primary tumor, left and right sampled lymph nodes **(B)**.

The patient is a 59-year-old male with clinically localized muscle-invasive urothelial bladder carcinoma who underwent radical cystoprostatectomy and bilateral lymph node dissection. Neoadjuvant chemotherapy was not administered due to renal insufficiency and significant gross hematuria. Fresh, surgically-resected tissue was obtained from four distinct bladder tumor regions and two representative lymph nodes from the right and left iliac fossae, and the tissue was dissociated into single cells for fluorescence activated cell sorting (Figure 1A). Final pathologic diagnosis was pT4 N0 with prostate stroma invasion and 43 negative lymph nodes, blindly reviewed by two independent genitourinary pathologists (J.S. and A.S.) with sample histology depicted (Figure 1B). Fluorescence activated cell sorting (Supplementary Figure 1A) was performed to isolate and quantify EPCAM^+^CD44^+^CD49f^+^ cells, with EPCAM staining incorporated to distinguish against non-epithelial cells [9, 11]. While sorted cell populations ranged from 0.04% to 0.27% as expected in the four primary tumor regions, we detected a smaller proportion of EPCAM^+^CD44^+^CD49f^+^ cells in the lymph nodes ranging from 0.002% to 0.004% (Supplementary Figure 1B).

**Figure 2:**
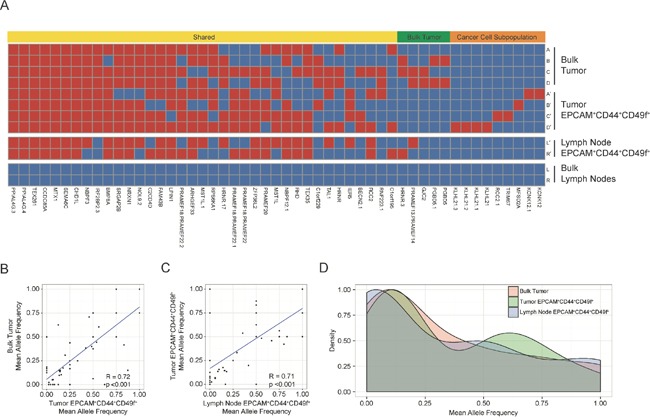
Distribution of somatic variants and allele frequencies The heatmap **(A)** shows regional distribution among all samples of 51 somatic variants present in all tumor and cancer cell subpopulations. The bulk tumor regions (samples A-D) and lymph nodes (samples L and R) and the corresponding EPCAM^+^CD44^+^CD49f^+^ subpopulations (denoted with′) are represented in the heatmap, and the presence of a variant is depicted as red while the absence of a variant is depicted as blue. Variants were categorized as shared (present in both bulk tumor and EPCAM^+^CD44^+^CD49f^+^ subpopulation samples), as bulk tumor (present in bulk tumor regions only), or as cancer cell subpopulation (present in EPCAM^+^CD44^+^CD49f^+^ subpopulations only). The scatter plots depict mean allele frequencies of somatic variants comparing bulk tumor samples and tumor subpopulations (p <0.001, Welch's unequal variances t-test) **(B)** as well as comparing tumor and lymph node EPCAM^+^CD44^+^CD49f^+^ subpopulations (p <0.001, Welch's unequal variances t-test) **(C)**. The histogram shows mean allele frequencies of the bulk tumor, tumor EPCAM^+^CD44^+^CD49f^+^ subpopulations, and lymph node EPCAM^+^CD44^+^CD49f^+^ subpopulations **(D)**.

To verify that the EPCAM^+^CD44^+^CD49f^+^ cells isolated from the lymph nodes were cancer cells derived from the primary tumor as well as to examine intratumoral genetic diversity, we performed whole-exome sequencing of bulk tumor samples, lymph nodes, and EPCAM^+^CD44^+^CD49f^+^ cells from each region generating 1,931,478,798 total reads which were aligned to the UCSC hg19 reference genome. Single nucleotide variants were analyzed and a total of 51 somatic variants were identified in tumor and EPCAM^+^CD44^+^CD49f^+^ samples compared to total lymph nodes, and of these we identified 20 synonymous variants, 26 non-synonymous variants, 3 insertion-deletions, and 2 unknown variants. Somatic variants present in both the bulk tumor and tumor EPCAM^+^CD44^+^CD49f^+^ subpopulations were categorized as shared (37/51, 72.5%), enriched in the bulk tumor (5/51, 9.8%), or enriched in the tumor EPCAM^+^CD44^+^CD49f^+^ cells (9/51, 17.6%) (Figure 2A). None of the variants present in the lymph node EPCAM^+^CD44^+^CD49f^+^ cells were exclusive as they were present in at least two other tumor or primary tumor EPCAM^+^CD44^+^CD49f^+^ samples. Allele frequencies of somatic single nucleotide variants were analyzed in order to further determine if EPCAM^+^CD44^+^CD49f^+^ cells isolated from the lymph nodes were analogous to EPCAM^+^CD44^+^CD49f^+^ cells isolated from the primary tumor. Scatter plots of allele frequencies demonstrate a significant correlation between bulk tumor and tumor EPCAM^+^CD44^+^CD49f^+^ cells somatic variant allele frequencies (R = 0.72, p < 0.001) (Figure 2B), between tumor EPCAM^+^CD44^+^CD49f^+^ cells and lymph node EPCAM^+^CD44^+^CD49f^+^ cells somatic variant allele frequencies (R = 0.71, p < 0.001) (Figure 2C), while bulk lymph nodes demonstrated an absence (allele frequency < 0.10) of the variants present in the primary tumor or EPCAM^+^CD44^+^CD49f^+^ cells (Supplementary Figure 2A and Supplementary Figure 2B). Histogram depiction of allele frequencies for bulk tumor, tumor EPCAM^+^CD44^+^CD49f^+^ cells, and lymph node EPCAM^+^CD44^+^CD49f^+^ cells illustrates the similarity among these fractions (Figure 2D). While the majority of the somatic variants identified in our analysis were shared, there was also evidence of clonal evolution in bulk tumors, represented by the variants enriched in the bulk tumor. Although unique variants were observed in the EPCAM^+^CD44^+^CD49f^+^ cells, these may correspond to a quiescent subpopulation of cancer initiating cells or represent *de novo* acquired mutations that have yet to populate the bulk tumor, as enrichment of the EPCAM^+^CD44^+^CD49f^+^ subpopulation may have uncovered variants that could be present in low frequency in the bulk tumor, but at higher frequency with respect to the cancer initiating cell subpopulation.

**Figure 3:**
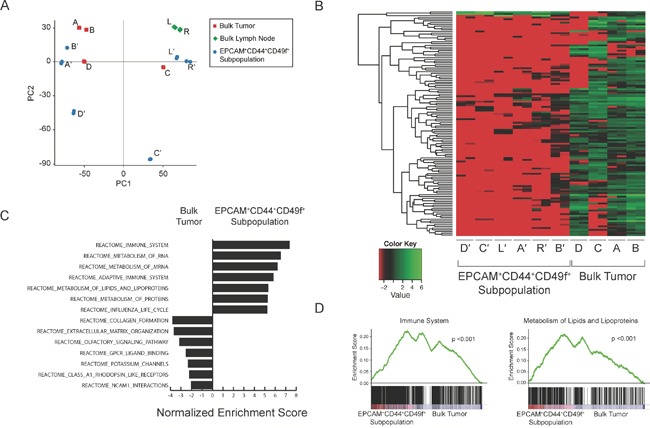
RNA-sequencing analysis of tumor and cancer cell subpopulations RNA-sequencing was performed with technical replicates (n=2). Principal component analysis plot of bulk tumor regions (red, samples A-D), lymph nodes (green, samples L and R), and EPCAM^+^CD44^+^CD49f^+^ subpopulations (blue, denoted with′) **(A)**. Heatmap depicts 123 genes differentially expressed between the bulk tumor regions and corresponding cancer cell subpopulations by DESeq and edgeR **(B)**. Reactome gene set enrichment analysis with the top 8 gene sets enriched in the EPCAM^+^CD44^+^CD49f^+^ subpopulations and bulk tumor regions listed (p <0.001 and p <0.05, respectively; FDR q-value < 0.001 and < 0.05, respectively; Kolmogorov-Smirnov statistic) **(C)** as well as representative plots demonstrating enrichment of immune system and lipid metabolism gene sets in cancer cell subpopulations (p <0.001, FDR q-value <0.001, Kolmogorov-Smirnov statistic) **(D)**.

To provide insight into the mechanisms of metastases, we performed RNA-sequencing to elucidate gene expression profiles unique to EPCAM^+^CD44^+^CD49f^+^. Despite genomic similarity between the bulk tumor and the EPCAM^+^CD44^+^CD49f^+^ cells, principal component analysis demonstrated heterogeneity among bulk tumor regions, lymph nodes, as well as the derived EPCAM^+^CD44^+^CD49f^+^ subpopulations (Figure 3A). Differential gene expression analysis generated 128 genes that segregated bulk tumor regions from EPCAM^+^CD44^+^CD49f^+^ cells (Figure 3B). Oncogene gene set enrichment analysis comparing the EPCAM^+^CD44^+^CD49f^+^ cells to the bulk tumor regions enriched for gene sets related to PTEN, KRAS, and BRCA1 in the bulk tumor, while gene sets associated with KRAS synthetic lethality (STK33 and TBK1), differentiation (HOXA9 and RPS14), and cAMP signaling were enriched in EPCAM^+^CD44^+^CD49f^+^ cells (Supplementary Figure 3). Both compartments enriched for gene sets related to RB. Reactome gene set enrichment analysis demonstrated statistically significant (p-value <0.001, FDR q-value <0.001) enrichment of gene sets related to lipid metabolism and the immune system in EPCAM^+^CD44^+^CD49f^+^ cells (Figure 3C and 3D). Gene expression in tumor EPCAM^+^CD44^+^CD49f^+^ cells further segregated intratumoral from lymph node EPCAM^+^CD44^+^CD49f^+^ cells with 381 genes differentially expressed, including upregulation of genes involved in immune responses and cell adhesion (Gene Ontology) in lymph node EPCAM^+^CD44^+^CD49f^+^ cells (Supplementary Figure 4).

## DISCUSSION

In this report we utilized a next-generation sequencing-based approach to both detect and quantify EPCAM^+^CD44^+^CD49f^+^ cells in surgically resected lymph nodes with a somatic variant profile similar to the primary tumor and tumor EPCAM^+^CD44^+^CD49f^+^ cells, providing evidence for the presence of micrometastatic disease despite negative traditional histologic staging. Using whole-exome sequencing we were unable to detect tumor variants in the bulk lymph nodes, however a key enrichment step for the EPCAM^+^CD44^+^CD49f^+^ subpopulation through fluorescence activated cell sorting provided the sensitivity needed to identify the presence of micrometastases in the lymph nodes. In the future, perhaps sorting for the more abundant EPCAM^+^ population may improve sensitivity for cancer detection. While previous studies in other cancers have identified micrometastases using RT-PCR, direct Sanger sequencing of specific genes, or histology, our study is the first, to our knowledge, in which whole-exome sequencing has been utilized to prospectively detect and quantify micrometastases in cancer [4, 12–14]. Furthermore, while intratumoral heterogeneity has previously been studied in urothelial bladder cancer on a chromosomal level, our analysis is the first to examine this using next-generation sequencing [15].

Enrichment for gene sets related to lipid metabolism and the immune system in the EPCAM^+^CD44^+^CD49f^+^ cells compared to the bulk tumor population, which included tumor-infiltrating and stromal cells, may represent pathways unique to EPCAM^+^CD44^+^CD49f^+^ cells. Lipid metabolism may play a central role in cancer, as *in vitro* inhibition of these pathways has been shown to influence cellular transformation, growth, and invasion [16]. Recently, targeting fatty acid oxidation has shown decreased tumor growth in an *in vivo* model of MYC-driven triple negative breast cancer [17]. Inflammatory pathways have shown both anti-tumor as well as a tumor promoting roles with evidence suggesting that inflammation may function to maintain cancer initiating cell populations [18]. Prostaglandin E-2 has been shown to promote bladder CSC re-population in tumors after treatment with chemotherapy, and targeting this pathway with a cyclooxygenase-2 inhibitor attenuated chemotherapy resistance [19]. In addition, we identified a subset of genes important in the immune response and immune cell activation enriched only in the lymph node EPCAM^+^CD44^+^CD49f^+^ cell populations. These metabolic and immune pathways may provide clues to the treatment resistance and pluripotency of cancer initiating cells, as well as the mechanisms of tumor metastasis or differences in cell niche that will need to be exploited in future studies. A cautionary note is that the immune signature could be influenced by contaminating immune cells from lymph nodes.

Identification of otherwise undetected micrometastases may have important clinical implications in prognosis, sequence of therapy, and targeting cancer pathways. Although our patient ultimately was diagnosed with T4 N0 disease, the ability to improve staging through detection of lymph node micrometastases may allow us to predict recurrence risk and select patients for adjuvant therapies in patients of all T stages with clinically localized (N0) disease. Indeed, this patient recurred 12 months after surgery. While detection of specific driver mutations may be more time and cost effective, the heterogeneity of bladder cancer has been broadly demonstrated and there is compelling need to identify novel mutations that may be instrumental to development of micrometastasis and warrants our unbiased approach. Next-generation sequencing technologies are becoming increasingly logistically and financially accessible with our process from tissue procurement to analyzed data accomplishable in approximately 3 weeks. Major limitations to our study include the report of a single patient and the lack of functional evidence as our archival tissue experienced low viability when thawed with the inability of even total tumor cells to generate xenografts. However, multiple studies have demonstrated how a genome sequencing based case report can still provide valuable information that can guide further clinical study [20, 21]. In the future, we will need to prospectively analyze a larger cohort of patients with a broad spectrum of clinical disease to test this approach before we can generalize our findings.

## MATERIALS AND METHODS

### Study approval

Written informed consent was obtained from the patient in this study through an Institutional Review Board approved protocol #11-001363 prior to inclusion in this study.

### Tissue acquisition

Fresh tissue from four distinct bladder tumor regions and two representative lymph nodes in the left and right iliac fossae were obtained from a single patient. Prior to bladder resection, the lymph nodes were dissected and sequestered from the primary tumor to prevent contamination. A cross section of the lymph nodes was utilized for our analysis while the remainder was returned for pathology. Tissue was dissociated into single cell suspensions and stained with antibodies for EPCAM (BD 347197), CD44 (BD 55479), and CD49f (BioLegend 313621) for fluorescence activated cell sorting (BD FACSAria) of cancer cell subpopulations [9]. DNA and RNA were isolated using a Qiagen All Prep DNA and RNA isolation kit. Bladder and lymph nodes underwent standard pathologic assessment including gross and histologic examination. Lymph nodes were trimmed of fat and embedded entirely. A single representative 4 to 6 μM section for each of 43 lymph nodes were obtained for hematoxylin and eosin staining.

### DNA sequencing and analysis

DNA libraries were generated (Nugen Ovation Ultralow) for whole-exome sequencing with paired-end sequencing (2×150 bp) and 250x coverage (Illumina HiSeq 3000). DNA sequencing reads were filtered for low quality scores, and aligned to the human genome (UCSC hg19) using BWA version 0.7.7. Read realignment, base quality score recalibration, and removal of duplicate reads were performed using GATK and Picard. Single nucleotide variations (SNV) and INDELs were independently detected by MuTect and SNVer.

### RNA sequencing and analysis

RNA quality was assessed (Agilent Bioanalyzer) and all RIN values were greater than 7.0. RNA libraries were generated (KAPA RNA) for RNA-sequencing with 50 bp paired-end sequencing in replicate (Illumina HiSeq 2500). Raw reads with low quality and reads containing adapters were filtered then mapped to the *Homo sapiens* reference genome (UCSC hg19) with the gapped aligner Tophat and only uniquely aligned reads were utilized. In total, 106,298,696 reads were uniquely aligned with an average mapability of 72% (Supplementary Figure 5A and Supplementary Figure 5B). Gene ontology enrichment analysis was performed using DAVID Bioinformatics Resources 6.7 (https://david.ncifcrf.gov/). Gene Set Enrichment Analysis was conducted with GSEA v2.2.0 using the MSigDB database v5.1 for CP:REACTOME: Reactome gene sets, and C6: oncogenic signatures.

### Statistics

A principal component analysis (PCA) was performed on normalized read counts of all samples using the 'prcomp' function in R. In this analysis, we selected 500 top ranked genes based on their variations across all samples using the function ‘rowVars’ in R matrixStats package. The histogram plot was generated using ggplot2 package and p-values corresponding to allele frequencies were derived from Welch's unequal variances *t*-test. The Kolmogorov-Smirnov statistic was utilized to obtain GSEA statistics as previously described [22].

## SUPPLEMENTARY FIGURES



## Abbreviations

CSC: cancer stem cells
GSEA: gene set enrichment analysis
PCA: principle component analysis
SNV: single nucleotide variations

## References

[1] Hautmann RE, de Petriconi RC, Pfeiffer C, Volkmer BG (2012). Radical cystectomy for urothelial carcinoma of the bladder without neoadjuvant or adjuvant therapy: long-term results in 1100 patients. Eur Urol.

[2] Powles T, Eder JP, Fine GD, Braiteh FS, Loriot Y, Cruz C, Bellmunt J, Burris HA, Petrylak DP, Teng SL, Shen X, Boyd Z, Hegde PS (2014). MPDL3280A (anti-PD-L1) treatment leads to clinical activity in metastatic bladder cancer. Nature.

[3] Sternberg CN, Skoneczna I, Kerst JM, Albers P, Fossa SD, Agerbaek M, Dumez H, de Santis M, Théodore C, Leahy MG, Chester JD, Verbaeys A, Daugaard G (2015). Immediate versus deferred chemotherapy after radical cystectomy in patients with pT3-pT4 or N+ M0 urothelial carcinoma of the bladder (EORTC 30994): an intergroup, open-label, randomised phase 3 trial. Lancet Oncol.

[4] Kurahashi T, Hara I, Oka N, Kamidono S, Eto H, Miyake H (2005). Detection of micrometastases in pelvic lymph nodes in patients undergoing radical cystectomy for locally invasive bladder cancer by real-time reverse transcriptase-PCR for cytokeratin 19 and uroplakin II. Clin Cancer Res.

[5] Kreso A, Dick JE (2014). Evolution of the cancer stem cell model. Cell Stem Cell.

[6] Lawson DA, Bhakta NR, Kessenbrock K, Prummel KD, Yu Y, Takai K, Zhou A, Eyob H, Balakrishnan S, Wang CY, Yaswen P, Goga A, Werb Z (2015). Single-cell analysis reveals a stem-cell program in human metastatic breast cancer cells. Nature.

[7] Smith BA, Sokolov A, Uzunangelov V, Baertsch R, Newton Y, Graim K, Mathis C, Cheng D, Stuart JM, Witte ON (2015). A basal stem cell signature identifies aggressive prostate cancer phenotypes. Proc Natl Acad Sci USA.

[8] Peek EM, Li DR, Zhang H, Kim HP, Zhang B, Garraway IP, Chin AI (2012). Stromal modulation of bladder cancer-initiating cells in a subcutaneous tumor model. Am J Cancer Res.

[9] Chan KS, Espinosa I, Chao M, Wong D, Ailles L, Diehn M, Gill H, Presti J, Chang HY, van de Rijn M, Shortliffe L, Weissman IL (2009). Identification, molecular characterization, clinical prognosis, and therapeutic targeting of human bladder tumor-initiating cells. Proc Natl Acad Sci USA.

[10] Volkmer JP, Sahoo D, Chin RK, Ho PL, Tang C, Kurtova AV, Willingham SB, Pazhanisamy SK, Contreras-Trujillo H, Storm TA, Lotan Y, Beck AH, Chung BI (2012). Three differentiation states risk-stratify bladder cancer into distinct subtypes. Proc Natl Acad Sci USA.

[11] Brunner A, Prelog M, Verdorfer I, Tzankov A, Mikuz G, Ensinger C (2008). EpCAM is predominantly expressed in high grade and advanced stage urothelial carcinoma of the bladder. J Clin Pathol.

[12] Klebig F, Fischer C, Petri S, Gerull H, Wagener C, Tschentscher P (2007). Limitations in molecular detection of lymph node micrometastasis from colorectal cancer. Diagn Mol Pathol.

[13] Morton DL, Thompson JF, Cochran AJ, Mozzillo N, Elashoff R, Essner R, Nieweg OE, Roses DF, Hoekstra HJ, Karakousis CP, Reintgen DS, Coventry BJ, Glass EC (2006). Sentinel-node biopsy or nodal observation in melanoma. N Engl J Med.

[14] Braun S, Vogl FD, Naume B, Janni W, Osborne MP, Coombes RC, Schlimok G, Diel IJ, Gerber B, Gebauer G, Pierga JY, Marth C, Oruzio D (2005). A pooled analysis of bone marrow micrometastasis in breast cancer. N Engl J Med.

[15] Conconi D, Panzeri E, Redaelli S, Bovo G, Viganò P, Strada G, Dalprà L, Bentivegna A (2014). Chromosomal imbalances in human bladder urothelial carcinoma: similarities and differences between biopsy samples and cancer stem-like cells. BMC Cancer.

[16] Hirsch HA, Iliopoulos D, Joshi A, Zhang Y, Jaeger SA, Bulyk M, Tsichlis PN, Shirley Liu X, Struhl K (2010). A transcriptional signature and common gene networks link cancer with lipid metabolism and diverse human diseases. Cancer Cell.

[17] Camarda R, Zhou AY, Kohnz RA, Balakrishnan S, Mahieu C, Anderton B, Eyob H, Kajimura S, Tward A, Krings G, Nomura DK, Goga A (2016). Inhibition of fatty acid oxidation as a therapy for MYC-overexpressing triple-negative breast cancer. Nat Med.

[18] Baron JA, Cole BF, Sandler RS, Haile RW, Ahnen D, Bresalier R, McKeown-Eyssen G, Summers RW, Rothstein R, Burke CA, Snover DC, Church TR, Allen JI (2003). A randomized trial of aspirin to prevent colorectal adenomas. N Engl J Med.

[19] Kurtova AV, Xiao J, Mo Q, Pazhanisamy S, Krasnow R, Lerner SP, Chen F, Roh TT, Lay E, Ho PL, Chan KS (2015). Blocking PGE2-induced tumour repopulation abrogates bladder cancer chemoresistance. Nature.

[20] Haffner MC, Mosbruger T, Esopi DM, Fedor H, Heaphy CM, Walker DA, Adejola N, Gürel M, Hicks J, Meeker AK, Halushka MK, Simons JW, Isaacs WB (2013). Tracking the clonal origin of lethal prostate cancer. J Clin Invest.

[21] Brannon AR, Sawyers CL (2013). “N of 1” case reports in the era of whole-genome sequencing. J Clin Invest.

[22] Subramanian A, Tamayo P, Mootha VK, Mukherjee S, Ebert BL, Gillette MA, Paulovich A, Pomeroy SL, Golub TR, Lander ES, Mesirov JP (2005). Gene set enrichment analysis: a knowledge-based approach for interpreting genome-wide expression profiles. Proc Natl Acad Sci USA.

