# Supplementary material for: Sequencing of cancer cell subpopulations identifies micrometastases in a bladder cancer patient
Source: Oncotarget. 2017 Apr 21;8(28):45619–25. doi: 10.18632/oncotarget.17312 (PMC5542213; doi:10.18632/oncotarget.17312)
Supplement: Supplementary file 1 [file oncotarget-08-45619-s001.pdf]

Sequencing of cancer cell subpopulations identifies micrometastases in a bladder cancer patient

SUPPLEMENTARY FIGURES

A

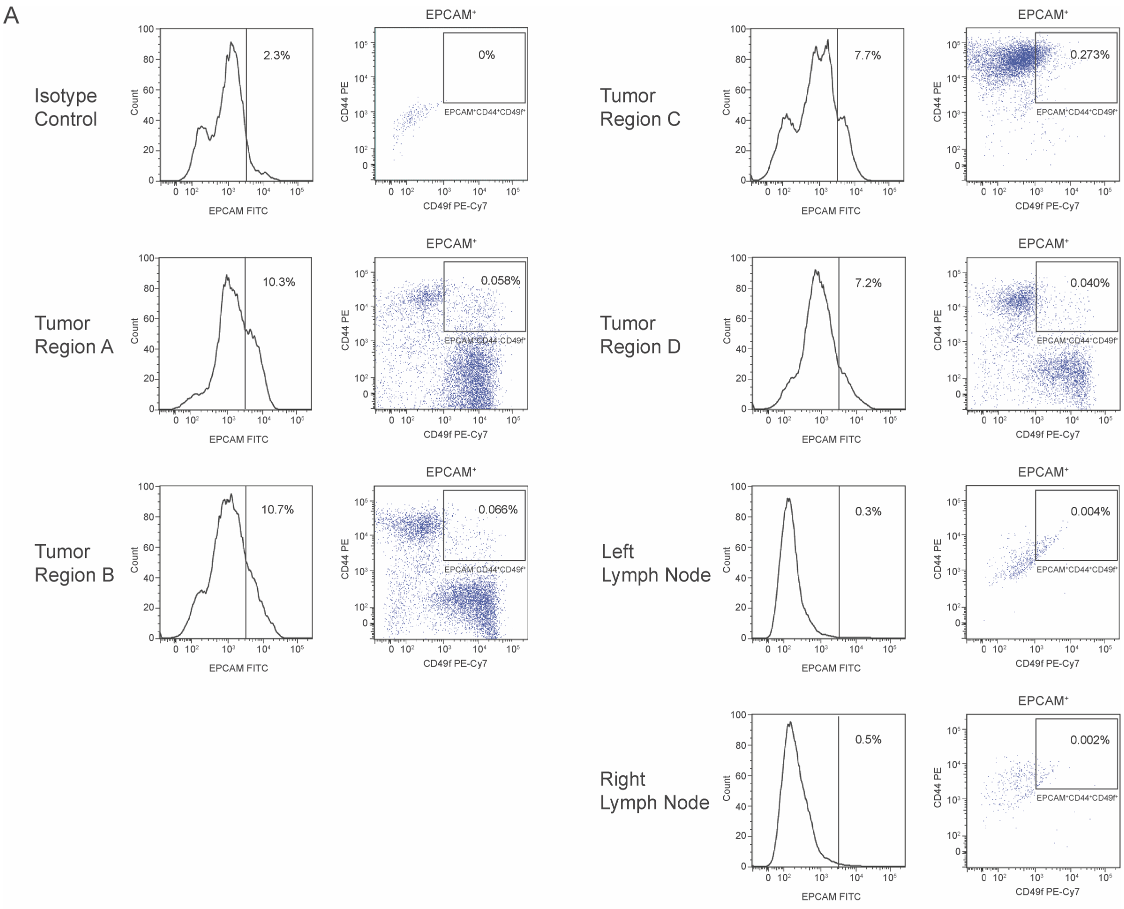

B

Region	Total Cells Sorted (x10^6)	EPCAM$^+$CD44$^+$CD49f$^+$ (%)
A	2.49	0.07
B	3.22	0.22
C	0.68	0.50
D	3.51	0.21
L	11.41	0.01
R	15.11	0.05

Supplementary Figure 1: Fluorescence Activated Cell Sorting of the Bladder Tumor and Lymph Nodes. Fresh tissue from four regions of the bladder tumor (Tumor Regions A-D) as well as the left and right lymph nodes were stained for EPCAM FITC, CD44 PE, and CD49f PE-Cy7, and fluorescence activated cell sorting was performed to isolate EPCAM+CD44+CD49f+ cancer cell subpopulations, and quantify percent EPCAM+ (histogram plots) and EPCAM+CD44+CD49f+ subpopulations (scatter plots) calculated as percent of the total number of cells sorted **(A)**. The table lists total cells sorted from each region and percentage of EPCAM+CD44+CD49f+ subpopulations **(B)**.

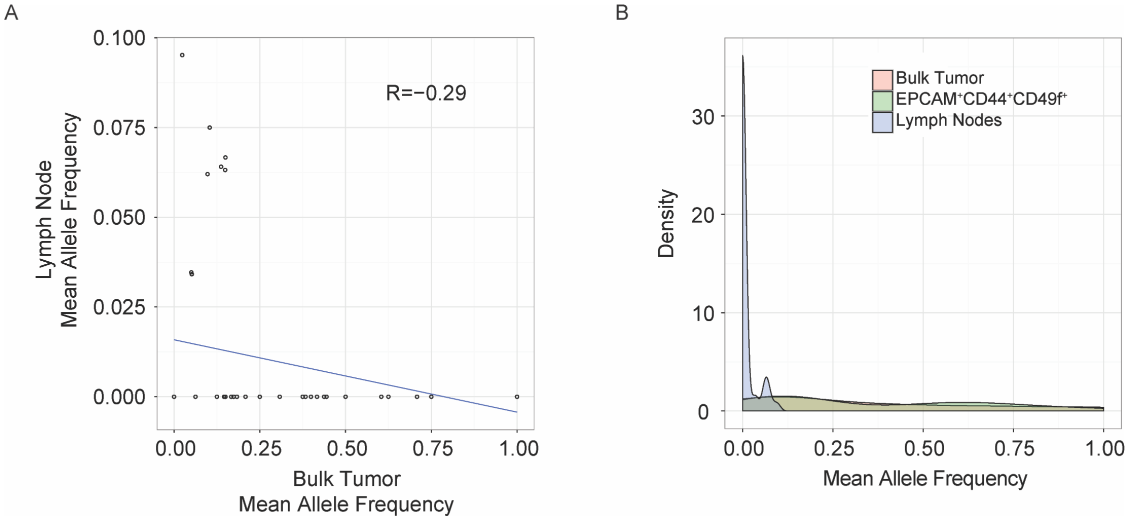

Supplementary Figure 2: Mean Allele Frequency of Lymph Nodes. The scatter plot depicts mean allele frequencies of somatic variants comparing the bulk lymph node to the bulk tumor **(A)**. The histogram depicts mean allele frequencies comparing the lymph nodes, the bulk tumor, and EPCAM+CD44+CD49f+ subpopulations **(B)**.

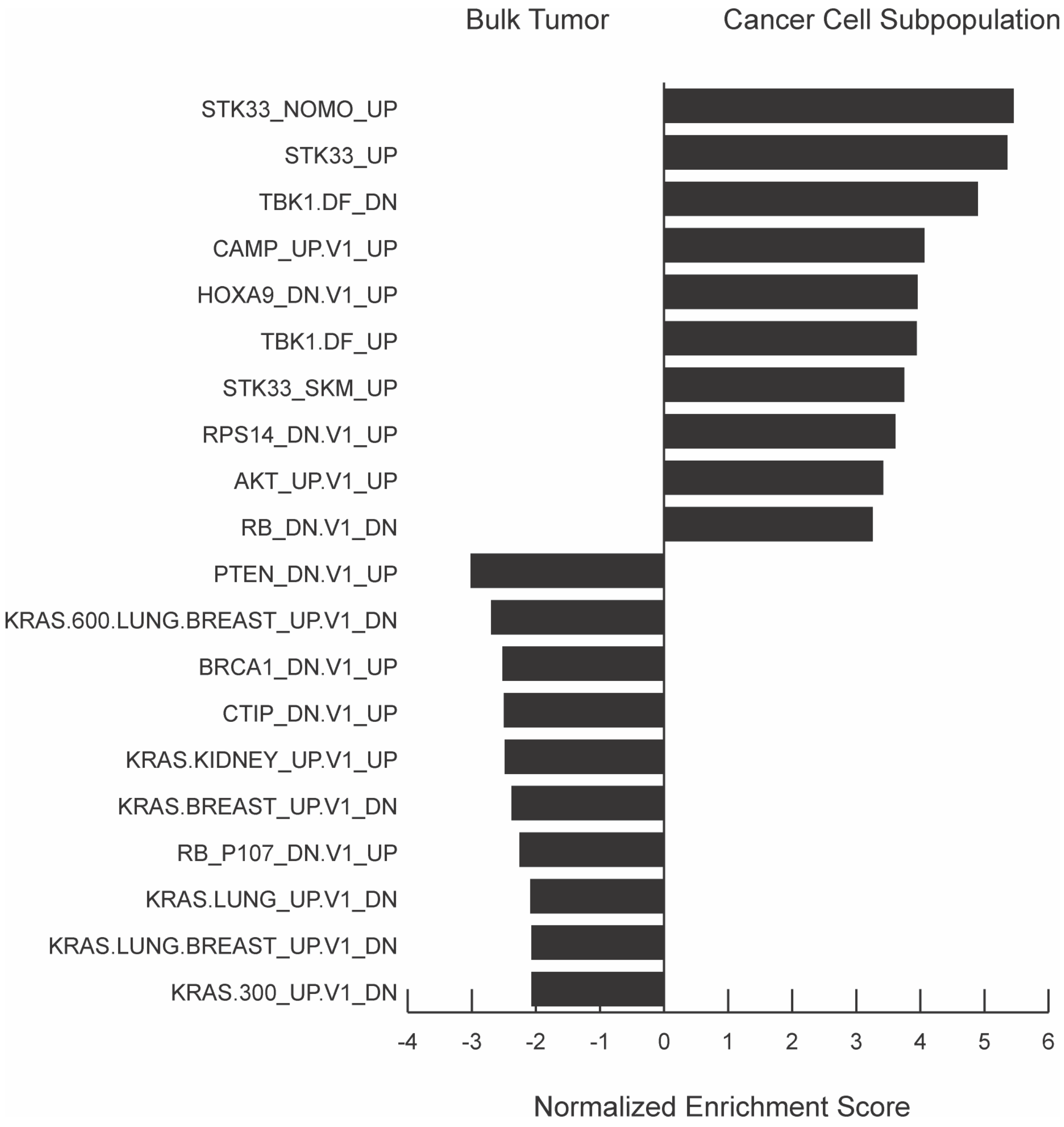

Supplementary Figure 3: Oncogene Gene Set Enrichment Analysis Comparing the Bulk Tumor to the EPCAM+CD44+CD49f+ Subpopulation. The top 10 oncogene gene sets (cancer stem cell FDR q-value < 0.001, bulk tumor FDR q-value < 0.05, Kolmogorov-Smirnov statistic) enriched in the bulk tumor regions and EPCAM+CD44+CD49f+ subpopulations.

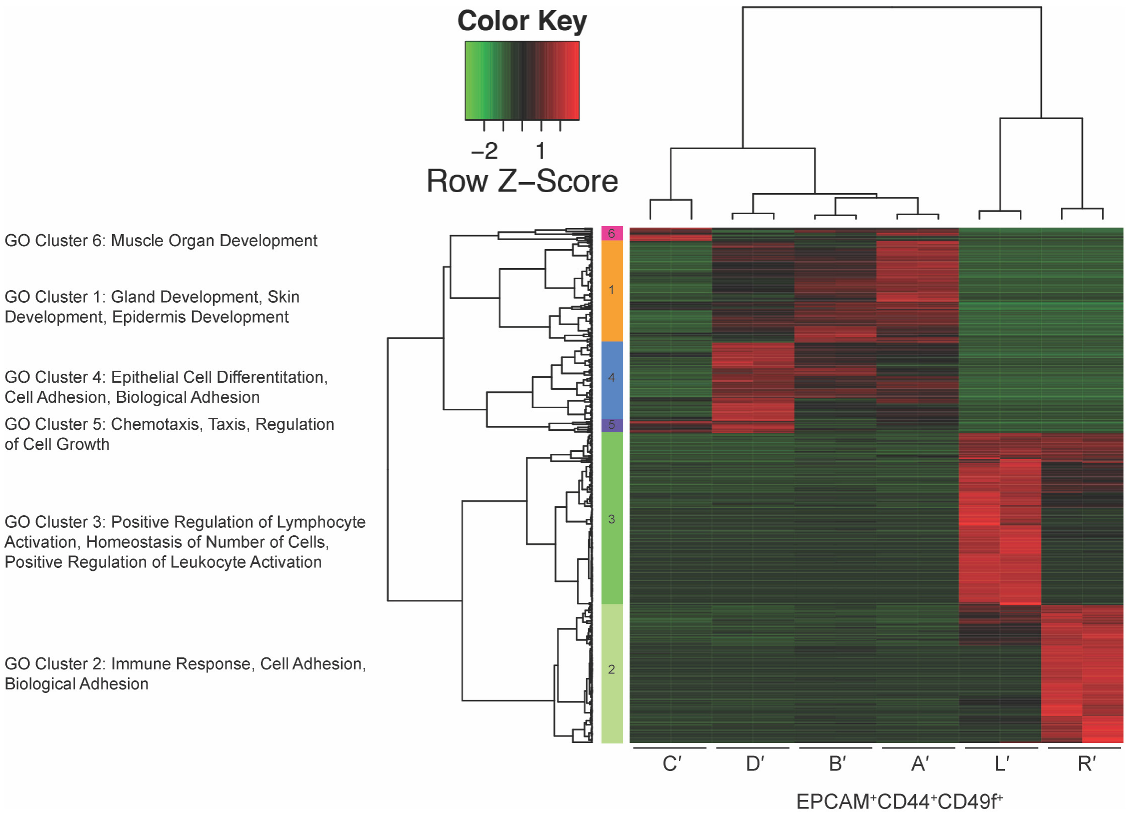

Supplementary Figure 4: Differential Gene Expression Comparing EPCAM+CD44+CD49f+ Subpopulations in Tumor Regions Versus EPCAM+CD44+CD49f+ Subpopulations in Lymph Nodes. The heatmap depicts 381 genes differentially expressed between EPCAM+CD44+CD49f+ subpopulations (denoted with′) in the primary tumor regions (A′-D′) versus the lymph nodes (L′ and R′). The top three Gene Ontology (GO) Terms associated with the clusters highlighted are listed to the left of the heatmap.

A

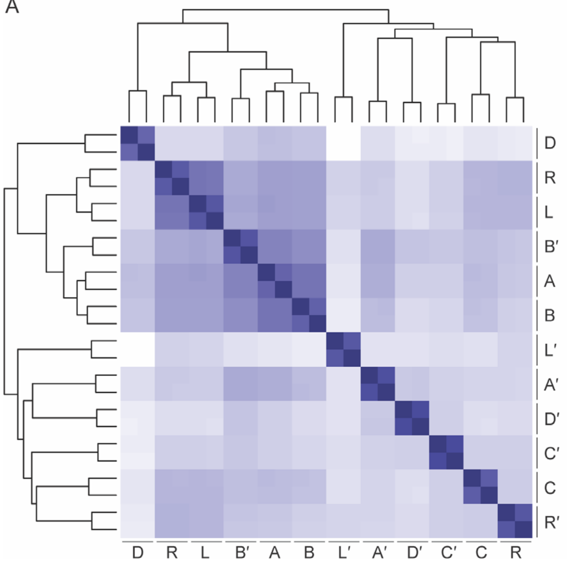

B

Sample	Number of Input Reads	Uniquely Mapped Reads Number	Uniquely Mapped Reads (%)
A	13216176	9867951	74.67%
A	12858575	9496307	73.85%
A′	13341857	9991205	74.89%
A′	12988084	9608282	73.98%
B	11893051	9820130	82.57%
B	11581419	9468583	81.76%
B′	13636884	9918403	72.73%
B′	13257982	9534556	71.92%
C	10093545	7346318	72.78%
C	9824138	7061536	71.88%
C′	13588067	9870572	72.64%
C′	13230958	9502690	71.82%
D	13531807	10677210	78.90%
D	13187297	10290934	78.04%
D′	12117971	8814231	72.74%
D′	11780071	8469906	71.90%
L	11827665	8151618	68.92%
L	11525815	7855333	68.15%
L′	11753510	7110328	60.50%
L′	11443294	6831753	59.70%
R	11558293	7673041	66.39%
R	11245472	7382815	65.65%
R′	10636242	7057689	66.36%
R′	10351961	6777524	65.47%

Supplementary Figure 5: RNA-Sequencing Correlation Plot and Alignment Statistics. RNA-Sequencing correlation plot **(A)** and alignments statistics **(B)** for technical duplicates of the bulk tumor regions (samples A-D), lymph nodes (samples L and R) and corresponding EPCAM+CD44+CD49f+ cancer cell subpopulations (denoted with′).
